# Molecular Pathology, Oxidative Stress, and Biomarkers in Obstructive Sleep Apnea

**DOI:** 10.3390/ijms24065478

**Published:** 2023-03-13

**Authors:** Piero Giuseppe Meliante, Federica Zoccali, Francesca Cascone, Vanessa Di Stefano, Antonio Greco, Marco de Vincentiis, Carla Petrella, Marco Fiore, Antonio Minni, Christian Barbato

**Affiliations:** 1 Department of Sense Organs DOS, Sapienza University of Rome, Viale del Policlinico 155, 00161 Roma, Italy; 2 Institute of Biochemistry and Cell Biology (IBBC), National Research Council (CNR), Department of Sense Organs DOS, Sapienza University of Rome, Viale del Policlinico 155, 00161 Roma, Italy; 3 Division of Otolaryngology-Head and Neck Surgery, Ospedale San Camillo de Lellis, ASL Rieti-Sapienza University, Viale Kennedy, 02100 Rieti, Italy

**Keywords:** obstructive sleep apnea syndrome, OSAS, OSA, oxidative stress, intermittent hypoxia, biomarkers, CPAP, cognitive

## Abstract

Obstructive sleep apnea syndrome (OSAS) is characterized by intermittent hypoxia (IH) during sleep due to recurrent upper airway obstruction. The derived oxidative stress (OS) leads to complications that do not only concern the sleep-wake rhythm but also systemic dysfunctions. The aim of this narrative literature review is to investigate molecular alterations, diagnostic markers, and potential medical therapies for OSAS. We analyzed the literature and synthesized the evidence collected. IH increases oxygen free radicals (ROS) and reduces antioxidant capacities. OS and metabolic alterations lead OSAS patients to undergo endothelial dysfunction, osteoporosis, systemic inflammation, increased cardiovascular risk, pulmonary remodeling, and neurological alterations. We treated molecular alterations known to date as useful for understanding the pathogenetic mechanisms and for their potential application as diagnostic markers. The most promising pharmacological therapies are those based on N-acetylcysteine (NAC), Vitamin C, Leptin, Dronabinol, or Atomoxetine + Oxybutynin, but all require further experimentation. CPAP remains the approved therapy capable of reversing most of the known molecular alterations; future drugs may be useful in treating the remaining dysfunctions.

## 1. Introduction

Obstructive sleep apnea syndrome (OSAS) affects from 9% to 39% of the adult population, with a higher incidence in males and the elderly, and is the most common form of respiratory sleep disorder [1,2,3,4]. It is characterized by recurrent, complete, or partial upper airway obstruction due to their collapse, with consequent hypopnea or apnea, leading to hypoventilation and chronic intermittent hypoxemia (IH) and increasing blood carbon dioxide partial pressure [5]. The OSAS consequences do not only concern excessive daytime sleepiness; they also independently favor the development of cardiovascular pathologies as an independent risk factor for hypercholesterolemia and hypertension, obesity, diabetes, and neuropsychological diseases such as depression [6,7,8,9,10,11,12]. Therefore, OSAS patients have higher cardiovascular-related morbidity compared with non-OSAS ones [13]. 

The diagnosis of OSAS follows the diagnostic criteria codified by the American Academy of Sleep Medicine (AASM) after the exclusion of other pathologies that may be the cause of the apnea/hypopnea events [14]. Furthermore, it is possible to make a diagnosis of OSAS when one is faced with anamnestic data, collected by interviewing the patient or those who sleep with him, with reported episodes of falling asleep when awake, excessive daytime sleepiness, non-refreshing sleep, tiredness, or insomnia, breathing, snoring, wheezing, choking, loud snoring, and at least 5 episodes of apnea, hypopnea, or breathing-related awakenings per hour in polysomnography. Alternatively, even in the absence of anamnestic data, it is possible to diagnose OSAS when polysomnography shows 15 or more apneas, hypopneas, or awakenings related to respiratory events per hour with evidence of respiratory effort in all or part of them in polysomnography [1]. To measure the degree of OSAS, the polysomnographic apnea-hypopnea index (AHI) is used [14,15]. Depending on the severity of the disease, treatment options include surgical interventions, lifestyle modifications, continuous positive airway pressure (CPAP), oral appliances such as mandibular advancement, and hypoglossal nerve stimulation [16,17,18]. However, the OSAS diagnosis and treatment currently do not take into consideration what happens at the molecular and cellular level, which instead causes systemic complications related to OSAS. This review aims to recognize molecular mechanisms, markers, and potential treatments useful for counteracting the cellular damage resulting from OSAS. This could help to better understand which patients are candidates, not only for the use of CPAP or MAD (mandibular advancement device), but also for medical therapy to counteract or reverse cellular damage.

## 2. Results

### 2.1. Oxidative Stress (OS)

OS is an imbalance between reactive oxygen species (ROS) production and antioxidant capacity. The hypoxia and reoxygenation cycles cause a change in the oxidative balance, leading to the formation of ROS and a decrease in endogenous antioxidant molecules [19]. ROS react with organic molecules, impairing their functions, altering cellular metabolism, and causing cell damage. They are considered one of the main mechanisms responsible for cardiovascular complications in patients with OSAS, and their production correlates with the severity of the disease [13,20,21,22].

The presence of oxidative imbalance has also been observed in uvular tissues removed after uvulopalatoplasty in patients with OSAS and correlates with the severity of the disease [23].

**Figure 1:**
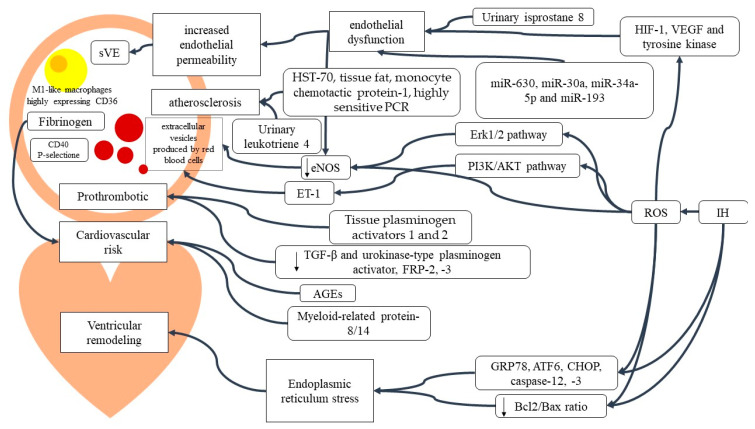
Mechanisms of cardiovascular risk in OSAS.

The human organism tries to adapt to the condition of lack of oxygen with the production of specific molecules useful for cell survival in hypoxic conditions such as Hypoxia Induced Factor-1α (HIF-1α) and Vascular endothelial growth factor (VEGF) [23,24]. HIF-1 α, instead, is one of the main actors in oxygen homeostasis. Its levels, together with those of NF-kB, correlate with the severity of the disease measured by the AHI and desaturation number, and the levels of surfactant protein D (SPD) are reduced in an inverse manner [24,25,26]. There is no significant variation in HIF-1α levels during the day [25,26]. The alteration of circulating HIF-1α levels is chronic; it has been observed that a single night with CPAP in severe OSAS patients does not significantly modify its expression [24]. The expression of HIF-1α and NF-kB is reduced after two months of continuous nasal CPAP use, and the SPD levels increase [25]. VEGF is increased as the nocturnal oxygen saturation decreases [24,25]. OS also underlies the increase in myeloperoxidase (MPO), intracellular adhesion molecule 1 (ICAM-1), vascular cell adhesion protein (VCAM-1), L-selectin, and E-selectin [26,27]. The differences in molecular expression in OSAS patients also concern the reduction of the morning levels of the Rho-associated protein kinase (ROCK) 1 and 2 molecules and the circulating nitric oxide, as well as the expression of endothelial eNOS [28,29]. These observations were confirmed in vivo, with an observed down-regulation of eNOS and an increase in nitrotyrosine [30]. VE-cadherin cleavage is one of the mechanisms of endothelial dysfunction and in OSAS patients, the circulating plasma values of the soluble form of VE-cadherin (sVE) were increased, suggesting an augmented endothelial permeability. This mechanism appears to be associated with ROS production, and activation of HIF-1, VEGF, and tyrosine kinase pathways [31]. Another mechanism that contributes to the endothelial dysfunction in IH is the production of extracellular vesicles by red blood cells, and their pathogenetic mechanism involves decreased eNOS, increased Endothelin-1 (ET-1) production via the Erk1/2 pathway, and phosphorylation via the PI3K/AKT pathway (Figure 1 [32]. OSAS-induced endothelial dysfunction has also been related to some microRNAs, such as miR-630 in infantile OSAS and miR-30a, miR-34a-5p, and miR-193 in mouse models (Figure 1) [33,34,35,36,37]. CPAP is widely used in the treatment of OSAS, and its systemic benefits have also been observed at the molecular level regarding endothelial function. Myocardial damage is also linked to microRNA expression alterations such as miR-146a-5p (Figure 1) [37]. 

#### 2.1.1. Mitochondrial Involvement

ROS are mainly produced at the mitochondrial level during the reoxygenation phase [27,38]. Mitochondrial damage is confirmed by the reduction of circulating mitochondrial DNA in conjunction with the worsening of OSAS [39]. Studies from mouse models showed that mitochondrial OS is also the basis of the damage to auditory hair cells, with aberrant mitochondrial morphology and overexpression of PGC-1α and Tfam mRNA [40]. The role of mitochondria in recurrent hypoxia damage is not limited to neuronal cells, and studies on genioglossus and palatine muscles have also shown damage at this level [41].

#### 2.1.2. Inflammatory Signaling

IH leads to the formation of proinflammatory factors such as tumor necrosis factor (TNF), C-reactive protein (CRP), and interleukins (IL)-6 and -8 [6,21,42,43], and elevated values of NF-kB and TNF-α are linked to OSAS and daytime sleepiness. Cytokines related to NF-kB are also consequently increased, such as IL-8 [42,44], whereas IL-17 levels are also correlated with OSAS and its severity, as well as an inverse correlation with Vitamin D levels in enrolled patients [45]. 

CRP and TNF-α levels decrease after OSAS surgery, but they still remain higher than those of healthy control groups [43].

Further molecules whose expression is altered are Osteoprotegerine, Chitinase 3-like protein 1 (YKL-40), and Cardiotrophin-1 (CT-1), and their value correlates with AHI [46].

#### 2.1.3. Peripheral Blood Cells

Various changes in peripheral blood cells have been observed. Overexpression of Toll-Like Receptor (TLR) was observed in circulating monocytes of OSAS patients [47], and the TLR 6 gene is upregulated in peripheral blood cells through DNA methylation. In particular, the cytosine-phosphate-guanine (CPG) site number 1 is hypermethylated in patients with severe OSAS, and its methylation is reduced after at least 6 months of CPAP therapy [48]. Epigenetic studies also identified hypermethylation of interleukin 1 receptor 2 (IL1R2) and androgen receptors with increased expression of both [49].

#### 2.1.4. Cardiovascular Implication of OSAS Inflammation

The role of inflammation has also been observed in the aortas of OSAS mouse models, evidencing an accumulation and proliferation of pro-inflammatory metabolic M1-like macrophages highly expressing CD36 and an increase in the transcription of atherogenic pathways, inflammation, and OS (Figure 1) [50]. Increased circulating fibrinogen values are associated with an elevated risk of cardiovascular events as well as OSAS, correlating high serum levels with the severity of the disease [51]. The inflammatory molecules increased in OSAS patients are numerous and also include heat shock protein 70, tissue fat, monocyte chemotactic protein-1, and highly sensitive C-reactive protein [52]. The prothrombotic state due to OSAS is also documented by the increase in tissue plasminogen activators 1 and 2 and the decrease in TGF-β and urokinase-type plasminogen activator [53]. Epigenetic dysregulation of DNA in OSAS was shown by aberrant methylation of the formyl peptide receptor (FPR) 1, 2, 3 genes, and FPR1 overexpression and the deficiency of FPR2 and FPR3 were associated with OSAS and its severity as well as with the development of diabetes mellitus and cardiovascular diseases.

Literature evidence did not show a correlation between OS and cardiovascular risk in OSAS patients [54].

Neurotrophins are proteins that regulate the nervous system; alterations of some of them, such as brain-derived neurotrophic factor (BDNF) and nerve growth factor (NGF), can also lead to cardiovascular complications. BDNF is related to cardiomyocyte contractility and its alterations with atherosclerosis and hypertension, and NGF plays a role in atrial autonomic OSAS-induced atrial fibrillation [54].

### 2.2. Circulating Metabolites

Previous research has shown that OSAS induces an increase in the blood levels of endocannabinoids such as anandamide and ethanolamide, which are associated with an increase in blood pressure and cardiovascular risk [55]. Likewise, an increase in the blood levels of adenosine, epinephrine, norepinephrine, and aldosterone was also observed [56,57], as was an increase in retinoids, carotenoids, and tocopherol, which increase the susceptibility to vascular pathologies [58]. 

Levels of saturated fatty acids and n-3 fatty acids also correlate with sleep quality, duration, and rapid eye movements. An increase in lactic acid and some fatty acids such as arabinose, arabitol, cellulose, glyceraldehyde, and threitol has been observed in these patients [59], and metabolomic studies have also shown an increase in glutamic acid, deoxy sugar, arachidonic acid, phosphatidylethanolamine, sphingomyelin, and lysophosphatidylcholine. The metabolic alterations mentioned above seem to be attributable to the hypoxia induced by OSAS [60]. 

In addition to the metabolites, the regulatory metabolism molecules are also impaired in OSAS patients. OSAS is independently correlated with insulin resistance and fatty liver disease, and several genes involved in cholesterol metabolism were impaired, such as malic enzyme and acetyl coenzyme A (CoA) synthetase, or acetyl-CoA carboxylase, stearoyl-CoA desaturases 1 and mitochondrial glycerol- 3-phosphate acyltransferase [61]. OSAS also induces adipose tissue inflammation and dysfunction [62].

The circulating omentin expression increased in OSAS [63]. Moreover, OSAS patients have significantly increased levels of leptin, affecting sleep, ventilation, and upper airway defenses [64,65].

### 2.3. Urine Molecules

Urine analysis from OSAS patients revealed high concentrations of adrenaline, noradrenaline, and homocysteine, which are associated with increased cardiovascular risk, as well as leukotriene E4. Increased levels of homovanillic acid, a metabolite of dopamine, and 3-4-dihydroxyphenylacetic acid were also measured [29,66,67]. Urinary isoprostane 8, which is associated with daytime sleepiness and OSAS, as well as being an element of endothelial damage [68], and an increase in urinary leukotriene-4 has also been observed, which is related to atherosclerosis [69,70]. The significance of these altered urine metabolites in OSAS is unknown.

### 2.4. Neurotransmitters

Several neurotransmitters could play a role in the pathogenesis of the disease. Histamine is responsible for the state of arousal in the central nervous system. In studies on mouse models, it also seems to have a role in the neuromuscular transmission that occurs from the hypoglossal nerve to the genioglossus, both of which are altered in OSAS patients [71]. 

Starting from the observation that apneas increase during REM sleep, due to motor inhibition of the cervical-cephalic muscles, several authors investigated the potential mechanism of muscular inactivation. REM sleep-related upper airway collapse is due to a change in norepinephrine and serotonin secretion by cranial nerve XII. The secretory patterns and increased distribution of α1-adrenoceptors are impaired in mouse models of IH during sleep [72].

Loss of muscle tone leading to recurrent airway collapse appears to be related to reduced activity of pharyngeal motoneurons, also due to decreased stimulation of cholinergic acetylcholine receptors. This molecule, therefore, not only acts at the level of the central nervous system with different concentrations depending on whether we are in a state of sleep or wakefulness, but its action also varies at a peripheral level and could be one of the mechanisms of muscular hypotonia pharyngeal in OSAS patients [73]. Grace et al., observed that the G-protein-coupled inwardly rectifying potassium (GIRK) channels are also involved in muscular inactivation during REM sleep. Therefore, the authors hypothesized that the potential target to counteract the loss of muscle tone is a potassium channel, and to avoid having an undesirable systemic effect, they suggested the inwardly rectifying potassium channel Kir2.4, which is expressed almost exclusively in the motor nuclei of the cranial nerves. To date, there are no clinical studies with this hypothesis.

The brain neurotrophic factor (BDNF) is a neurotrophin responsible for neuron growth, development, and plasticity. Therefore, it has a role in memory and learning mechanisms. It can cross the brain-blood barrier, and its blood level dysregulation is related to depression and cognitive decline. Despite there being no significant difference in BDNF levels between OSAS and control patients, the morning molecule expression is related to age and oxygen saturation during sleep. The proBDNF, a derived product of BDNF, relates to age and HIF-1α morning quantity [74]. BDNF and proBDNF evening concentrations are higher in patients with alteration measured with the Athens Insomnia Scale (AIS), Pittsburg Sleep Quality Index (PSQUI) positive, and lower in those with Beck Depression Inventory (BDI) positive [75]. There is also a correlation between plasmatic BDNF levels and the oxygen desaturation index, and a negative one with the AHI. It has been hypothesized that BDNF could be involved in apoptosis-related neural injury, contributing to OSAS-induced cognitive degeneration and psychiatric pathologies [76]. OSAS patients with depression have lower levels of BDNF and pro-BDNF compared with OSAS patients with no mood disturbances [76].

IH could also be responsible for peripheric neural damage. In mice models, IH-induced OS decreases BDNF and pro-BDNF expression, which increases retinal cell apoptosis [72]. BDNF expression is altered in cognitive diseases (e.g., Alzheimer’s disease, Parkinson’s related dementia, etc.) [77] BDNF is also involved in nociception, and some authors have hypothesized his involvement in the greater pain susceptibility of OSAS patients compared with the general population [78].

Further neurotrophins are known and are related to OSAS, such as the nerve growth factor (NGF), neurotrophins 3 and 4, and one neurotrophic factor, the glial cell-line-derived neurotrophic factor (GDNF) [79]. NGF is responsible for sympathetic neurons’ maturation, differentiation, and survival. His precursor protein, the proNGF, has the opposite action [73]. Its neurological involvement in OSAS has not been observed, but it has been related to cardiac autonomic nervous system disturbances and pediatric neurogenic tonsillar inflammation and hypertrophy [54,79].

GDNF is essential for dopaminergic system development and respiratory pattern generation. It has been observed that its levels are lower in patients with OSAS [54,80].

OSAS exacerbates Parkinson’s disease; sleep disturbance severity and motor dysfunction have been related to IL-6 levels. In the same study, Kaminska et al. observed a correlation between BDNF and increased sleepiness [81].

#### OSAS, Neurocognition and Neurofilament

Oxidative stress has repercussions in many parts of the body, including the central nervous system. Therefore, OS in OSAS is also responsible for neurocognitive dysfunction. The imbalance between oxidization and antioxidants, as previously cited, could cause neuron injury in brain regions most susceptible to hypoxia and oxidative stress, such as the hippocampus and cerebral cortex regions [82]. Different domains could be altered in OSAS patients, such as attention/vigilance, memory, global cognitive, and executive function. However, CPAP treatment seems to improve some but not all executive functions in different degrees of cognitive dysfunction. Compromised cognition could be partially reversed after CPAP. As mentioned above, several studies highlighted the association between OS and nervous system diseases such as Parkinson’s disease, Alzheimer’s disease, and epilepsy [54]. In this direction, identification and quantification of neuronal biomarkers of axonal damage could improve the diagnostic accuracy and the prognostic assessment in the management of neurological disease. Neurofilament light chain (NFL) is a neuronal protein, exclusively located in the neuronal cytoplasm, whose levels increase in serum and cerebrospinal fluid (CSF) proportionally to the degree of neuronal axonal damage, and it is a valuable biomarker in several neurological disorders. In OSAS patients, it could be interesting to monitor NFL variations after CPAP treatment aimed to evaluate neuronal recovery. The neuronal damage has also been confirmed by the first studies, which show an increase in the expression of neurofilament (NFL) and its correlation with the severity of the disease [83]. Further studies are needed to measure the impact of CPAP on this parameter. 

### 2.5. Potential Therapies

#### 2.5.1. Antioxidants

There are many potentially beneficial molecules for patients with OSAS, but most of them have not been tested on humans. Manganese superoxide dismutase is protective against cortical neuron oxidative damage by IH in mouse models [84]. Adiponectin also proved to be useful in counteracting mitochondrial damage in the genioglossus muscle of OSAS mice [46].

In addition, ROS scavenger administration with Endavarone in mouse models of IH has been tested, showing a significant reduction in cognitive impairment associated with increased brain expression of phosphorylated-cAMP response element-binding (p-CREB) [85].

The molecules tested in humans have not been studied in courts large enough to give indications for their use. Vitamin C and N-acetylcysteine (NAC) have shown interesting results in the reduction of OS in OSAS [7], and NAC reduces OS in OSAS through the reduction of peroxidized lipids and the increase in glutathione. Surprisingly, patients who received it continuously also had improvements in sleep parameters [86]. Vitamin C, on the other hand, proved to be effective in improving the endothelial function of OSAS patients in a study that took as its reference the diameter of the brachial artery, an indirect indicator of endothelial function [87]. Lastly, Leptin is both a drug capable of reducing free radicals, OS, and atherosclerosis in patients with OSAS [88].

To date, the only confirmed antioxidant therapy is CPAP itself, which has shown to be able to reverse many of the molecular alterations observed in vivo, such as eNOS, nitro-tyrosine, and NF-kB in the endothelium and circulating TNF-α [32]. 

#### 2.5.2. Non-Antioxidant-Based Therapy

Estrogen-related receptor-α (ERR-α) is downregulated in OSAS patients and its ligand-binding induces the expression of fast-type muscle fibers in palatopharyngeal muscles. The interaction between estrogens and ERR-α could be a therapeutic target to reverse the muscle remodeling typical of these patients [89]. They inhibit the overexpression of HIF-1α induced by chronic IH and improve the endurance and regeneration of the genioglossus muscle in OSAS animal models [90]. Estrogens, in particular 17β-estradiol (E2) and a resveratrol dimer (RD), have a protective action against OSAS by limitation of HIF-1α action.

A pilot study in OSAS patients evidenced that Desipramine reduced airway collapse. At the same time, its anti-inflammatory properties could be beneficial in counteracting the systemic effects of OSAS, but further clinical studies are needed on a larger scale to evaluate its application [91,92].

The use of sedatives in the treatment of OSAS appears to be counterintuitive. However, it has been hypothesized that trazodone may reduce the respiratory arousal threshold and upper airway obstruction. The first phenomenon occurred in an experimental group, while the second was not significant, and the magnitude of the threshold change was not sufficient to counteract the changes due to mechanical obstruction [93].

The Phase II Pharmacotherapy of Apnea by Cannabimimetic Enhancement (PACE) has shown encouraging preliminary results for Dronabinol. A reduction in the AHI, a reduction in the feeling of sleepiness, and good satisfaction in the treated patients have been observed [94].

Sildenafil involves the inhibition of cyclin guanosine monophosphate phosphodiesterase 5, resulting in an increase in cyclic guanosine monophosphate and NO. Its experimentation in a randomized controlled trial in which it was compared with a placebo, however, showed a worsening of the disease [95].

In a randomized trial, the combination of Atomoxetine, a norepinephrine reuptake inhibitor, and antimuscarinic Oxybutynin, taken before going to sleep, was shown to be able to reduce the severity of OSAS, and further studies on larger sample sizes are needed [96].

It is important to observe that many molecules have only been tested in mouse models, such as Astragaloside IV, which showed an improvement of hypoxia-induced endothelial function [97]; Tauroursodeoxycholic acid, against hepatic damage induced by HI [98]; Pitavastatin, showing a reversal of IH-induced myocardial hypertrophy, cardiac function, perivascular fibrosis and inflammatory indices [99]; Allopurinol also showed beneficial effects in mouse models of OSAS with a reduction of lipid peroxidation and an improvement in cardiac function [100]; and for all molecules, clinical trials are needed.

### 2.6. Diagnostic Biomarkers

Some authors have attempted to identify OSAS biomarkers. From the studies of Fleming et al. it has emerged that altered values of glycated hemoglobin, c-reactive protein, and erythropoietin may be useful in OSAS diagnosis [101]. Variants of the 5-hydroxytryptamine receptor 2A have also been studied as markers of risk and severity of OSAS. It has been observed that some of them can be protective, while others predispose to greater severity of the disease [102]. 

The study of the overexpression of the genes disintegrin and metalloproteinase domain 29 (ADAM29), solute carrier family 18 (vesicular acetylcholine) member 3 (SLC18A3), and fibronectin-like domain-containing leucine-rich transmembrane protein 2 (FLRT2) showed how these latter are overexpressed in Asian subjects with OSAS and could be used to screen patients, at risk for the severe form of the disease [103].

Numerous genetic polymorphisms have been associated with the development of OSAS, but none of them have been validated in clinical trials as useful for screening or diagnosis [104,105,106,107,108,109,110,111].

MicroRNAs could be helpful for diagnosis, but we still don’t have clinical validations in OSAS [112,113,114], such as the downregulation of miR-664a-3p [115], and dysregulation of miR-126-3p, miR-26a-5p, and miR-107, which associate with arterial hypertension in OSAS patients [116]. (Table 1)

The most accurate OS markers in OSAS are thioredoxin, malondialdehyde, superoxide dismutase, and iron reduction [7]. Thioredoxin (TRX) concentration is a marker of disease severity as it is proportional to that of AHI and is inversely related to oxygen saturation [117,118].

Although several molecules related to OS were not significantly increased in OSAS, glutathione, 8-isoprostane, substances reactive to barbituric acid (TBARS), catalase activity, copper-zinc superoxide dismutase (SOD), and products of lipid peroxidation [119,120]. However, there is no agreement between SOD and malondialdehyde (a TBARS), because, in some studies, their levels seem to be correlated with the severity of OSAS [120,121]. The markers of the loss of antioxidant capacity in OSAS patients have been observed in several studies as the lowering of the antioxidant power of reduced iron (FRAP), the concentration of reduced iron, and the total serum antioxidant status (TAS) [122,123,124].

## 3. Discussion

Patients with OSAS often have an altered lipidic profile. The molecular imbalances above mentioned could determine alterations detectable before OSAS leads to the typical increase in total cholesterol, triglycerides, low-density lipoprotein, high-density lipoprotein, and low-density lipoprotein cholesterol [64]. The metabolic imbalance observed in the OSAS leading to an increase in glycolysis products seems to be also attributable to the action of HIF-1α which also induces glycolytic enzymes [125]. However, the list of impaired molecules in OSAS is wide, such as cardiolipin, phosphatidylcholine, phosphatidylethanolamine, bile acids, and oxylipids [126].

Since a correlation between OSAS and Vitamin D levels has been observed, its concentration measurement and possible supplementation could be useful, not to improve apneas but for the systemic damage connected to the deficiency [44].

IH causes increased production of ROS and a reduction of endogenous antioxidant molecules [19]. OS is a crucial component of dysfunctional pathologies associated with OSAS, such as obesity, hypertension, dyslipidemia, sympathetic activation, and diabetes. It has even been hypothesized that the OS produced by IH favors the development of obesity, thus favoring the development of OSAS [127]. It has been observed that the reduction of antioxidant enzymes occurs through the methylation of DNA, which is reversible with the normalization of breathing, inverting ROS production. Along with it, the chemosensory reflex of the carotid body and hypertension, which are impaired following IH, also stabilize [128].

Aldosterone levels increase with the increasing severity of OSAS. Aldosterone is a molecule related to resistant hypertension, just as OSAS is a disease related to the same disorder [57]. The interaction between the increase in all the proinflammatory molecules and the endothelium could be one of the causes of the increased cardiovascular risk in OSAS, such as TNF, interleukins, NF-kB, TLR receptors, myeloid-related protein-8/14, the accumulation of pro-inflammatory metabolic M1-lie macrophages highly expressing CD36, fibrinogen, shock protein 70, monocyte chemotactic protein 1, highly sensitive C-reactive protein, P-selectin, soluble CD40, ICAM-1, VCAM-1, L-selectin, E-selectin and MPO [6,20,42,43,44,45,48,51,52,55]. 

Some authors hypothesized the role of microRNAs (miR-126-3p, miR-26a-5p, and miR-107) in the diagnosis of arterial hypertension in OSAS patients [129]. In our opinion, they could be useful for better understanding the etiopathogenetic mechanisms, but they are certainly less relevant from a hypertension diagnostic point of view.

In the brain, chronic IH is associated with hippocampal cortical damage. The OS produced by the cycles of ischemia and reoxygenation with ROS production is thought to mimic that of stroke. Confirmation of this phenomenon was investigated in mouse models, and an increase in ROS and OS response markers was observed. Recurrent ischemia induced for prolonged times induces an increase in the molecules produced by the action of ROS, such as oxidized proteins, peroxidized lipids, and oxidized nucleic acids, with activation of caspase 3 and neuronal cell apoptosis. Further, confirming the role of OS in neuronal degeneration following OSAS, it was observed that mice overexpressing ROS scavenger molecules were less susceptible to neuronal damage following recurrent ischemia [19]. Oxidative damage from IH also affects areas of the sleep-wake rhythm, which could aggravate the persistent feeling of sleepiness [130]. Regarding the potential neurocognitive dysfunction induced by OSAS, it should also be noted that markers of Alzheimer’s disease (Aβ40, t-tau, p-tau) are increased in affected patients [131]. In the study of OSAS-related cognitive impairment, it was observed that it is associated with the expression of miR-26b and miR-207 [132]. 

The chronic IH that occurs in OSAS patients also leads to the formation of ROS at the cortical level, and their production increases above all during the re-oxygenation phases. ROS are considered responsible for cortical oxidative damage and the reduction of neurocognitive functions. The maximum production of ROS appears to occur at the mitochondrial level [38,83]. Mitochondrial OS is also responsible for damage to auditory sensory neuronal hair cells. The study of mouse models of hypoxia has highlighted alterations in mitochondrial morphology, and alterations in mRNA expression and leaves us with the prospect of experimenting with drugs active at this level to prevent hearing loss in OSAS patients [38]. It should be evaluated whether a mechanic counteracting airway collapse, associated with substances that reduce or reverse mitochondrial oxidative damage to cortical neurons, can alleviate neuronal damage. 

Several molecules are potentially useful in counteracting OS, cardiovascular, and neuronal damage due to IH, such as NAC, Vitamin C, Leptin, Dronabinol, and a combination of Atomoxetine and Oxybutinin [7,81,82,92,94]. 

From a clinical point of view, however, it is also useful to know which drugs are not suggested in patients suffering from a specific pathology. Indeed, even if the randomized trial on Sildenafil showed a negative impact on OSAS, the study is useful from a clinical point of view. Patients with OSAS suffer more frequently from erectile dysfunction; therefore, it is necessary to remember in their treatment not to administer this drug as it is pejorative for their disease [93].

The only confirmed antioxidant therapy is CPAP itself, which is able to reverse many of the molecular alterations [43]. Therefore, the utility of molecular antioxidant therapies must be viewed with caution. The mechanisms underlying cell damage could be disrupted by the restoration of normal nocturnal oxygenation through CPAP. This phenomenon has been observed, for example, with the return to normal values of eNOS, nitrotyrosine, and NF-kB in the endothelium of OSAS subjects after the use of CPAP and, as noted by Ryan et al., with TNF-α [44]. Similarly, CPAP also reduces silent brain infarctions, which are usually increased in subjects with OSAS. Therefore, also in this case, the mechanical action of the positive pressure and the rebalancing of nocturnal oxygenation are sufficient to interrupt the phenomenon [55]. CPAP rebalances the expression of numerous pro-inflammatory and pro-coagulation molecules, but it is not always able to restore the levels of healthy subjects. This is the case with inhibitors of plasminogen-1 activation and TGF-β [52].

Importantly, not all damages appear to be reversible with CPAP. Aortic injury mediated by pro-inflammatory metabolic M1-like macrophages highly expressing CD36, upregulation of atherogenic pathway transcription, inflammation, and OS in mouse models, once triggered, is not reversed by a return to normal oxygenation values [51].

Despite the identification of numerous molecules whose levels are significantly increased in patients with OSAS, studies that have tried to identify diagnostic or severity biomarkers have not led to conclusive results [27]. Plasma thioredoxin (TRX) is a marker of OS, and CPAP is able to reduce its concentration after 1 month of treatment [119]. In this direction, there are several molecules that can potentially support the clinician in selecting patients for specific antioxidant therapy, such as MMP-2, -9, highly sensitive C-reactive protein, soluble receptors for advanced glycation end-products (sRAGE), and copper (Cu).

We know that cellular hypoxia induces acidosis, but an interesting observation has also been made on the role that IH has on the ability of cells to respond to it. As an effect of IH, the overproduced ROS induces the release of Ca^2+^ and the entry of Na^+^ through the activation of the Na^+^/Ca^2+^ exchanger, resulting in an increase in Na^+^ ions that inhibit the activity of the Na^+^/H^+^ exchanger, leading to an accumulation of H^+^ ions and acidosis [133].

## 4. Materials and Methods

### 4.1. Literature Research

We performed a narrative literature review with articles from PubMed, Embase, and the Cochrane Central Register of Controlled Trials. We considered articles concerning molecular and metabolic alterations, OS, biomarkers, and antioxidant therapy in OSAS patients. We also analyzed the bibliography of the selected manuscripts for further relevant articles. 

### 4.2. Inclusion and Exclusion Criteria

We have considered articles in the English language without time limits. We preferred in vivo clinical analyses, but manuscripts describing animal models were not excluded if useful for understanding and completing the mechanisms analyzed. Unpublished studies were not considered for the present review. 

### 4.3. Data Selection

As this is a narrative review, the decision regarding the inclusion of each article was addressed jointly by the authors. After a careful selection of sources, the collected evidence was discussed by the authors and summarized in this manuscript.

## 5. Conclusions

OSAS is not only a pathology related to sleep dysfunction but also has a significant systemic impact. OS and IH lead to impaired endothelial function, osteoporosis, metabolic alterations, systemic inflammation, cardiovascular complications, central and peripheral neuronal degeneration, and pulmonary remodeling.

To counteract systemic effects, therapies based on NAC, Vitamin C, Leptin, or a combination of Dronabinol and Atomoxetine appear to have promising results. Currently, the only approved therapy is CPAP, which is also capable of reversing many of the observed molecular alterations. Therefore, drug therapy can be useful in the treatment of all those dysfunctions that remain even after the restoration of normal nocturnal oxygenation.

OSAS patients are more frequently subject to erectile dysfunction; however, Sildenafil should not be prescribed because it worsens the underlying disease. OSAS patients are also more prone to vitamin D deficiency; therefore, this should be sought in newly diagnosed patients and corrected.

In conclusion, it is important to discover new biomarkers through innovative diagnostic tools [134,135], opening a new translational phase aimed at tuning oxidative profiles in OSAS patients.

## Figures and Tables

**Table 1:** Potential OSAS diagnostic biomarkers. Clinical validations are needed.

Potential Biomarkers	References
glycated hemoglobin, c-reactive protein, erythropoietin	[101]
5-hydroxytryptamine receptor 2A	[102]
ADAM29, SLC18A3, FLRT2	[103]
miR-664a-3p, miR-126-3p, miR-26a-5p, miR-107	[115,116]
thioredoxin, malondialdehyde, superoxide dismutase	[117,118,119]
TBARS	[120,121]
FRAP	[122,123]
TAS	[124]

## Data Availability

Not applicable.
